# Specific associations of serum FGF23 and soluble Klotho with different types of left ventricular hypertrophy in hypertensive peritoneal dialysis patients: a cross-sectional study

**DOI:** 10.3389/fcvm.2025.1724067

**Published:** 2026-01-15

**Authors:** Yiyi Zhu, Xun Yin, Hong Zhang, Zhe Han, Jingjuan Lu, Muyuan Lin, Gang Chen, Yang Chen

**Affiliations:** Department of Nephrology, Changshu Second People’s Hospital, Changshu, Jiangsu, China

**Keywords:** fibroblast growth factor-23, left ventricular hypertrophy, peritoneal dialysis, restrictive cubic spline, soluble α-Klotho

## Abstract

**Objective:**

This study aimed to investigate the association between serum fibroblast growth factor 23 (FGF23) and soluble α-Klotho levels with left ventricular hypertrophy (LVH) in hypertensive patients undergoing peritoneal dialysis (PD). We also sought to evaluate their potential as biomarkers of left ventricular remodelling and to analyze potential non-linear relationships with the left ventricular mass index (LVMI), including subgroup differences.

**Methods:**

In this cross-sectional study, 124 hypertensive PD patients were enrolled. Serum concentrations of FGF23 and soluble α-Klotho were measured via enzyme-linked immunosorbent assay (ELISA). Echocardiography was used to assess left ventricular structure and define LVH. Multivariate logistic regression analysis was performed to evaluate the independent associations of these biomarkers with LVH. A restricted cubic spline (RCS) model was employed to explore non-linear relationships with LVMI.

**Results:**

The prevalence of LVH was 62.9%. After adjusting for gender, systolic blood pressure C–reactive, protein, haemoglobin, serum calcium, ejection fraction, serum phosphorus, and parathyroid hormone, multivariate analysis identified soluble α-Klotho as an independent protective factor against LVH (OR = 0.415, 95% CI: 0.247–0.643, *P* < 0.001), whereas FGF23 was an independent risk factor (OR = 1.260, 95% CI: 1.079–1.501, *P* = 0.005). RCS analysis revealed a significant non-linear relationship between FGF23 and LVMI (*P* < 0.001), with an inflection point at approximately 39.8 pg/mL. This association was more pronounced in patients aged >61 years and in males. The overall association between soluble α-Klotho and LVMI was not statistically significant.

**Conclusion:**

Among hypertensive PD patients, serum soluble α-Klotho is an independent protective biomarker for LVH, while elevated FGF23 levels are associated with an increased risk of LVH, suggesting an interaction between the two. FGF23 demonstrates a non-linear association with LVMI, which is modified by age and gender. Concurrent measurement of FGF23 and soluble α-Klotho may help identify patients at high risk for cardiovascular remodelling, thereby informing risk stratification and personalized management strategies.

## 1 Introduction

Cardiovascular disease (CVD) is the most common complication and the leading cause of mortality in patients with chronic kidney disease (CKD) (1, 2). Left ventricular hypertrophy (LVH) represents the most prevalent cardiac structural abnormality in this high-risk population and is a key precursor to heart failure (3, 4). Among patients with end-stage kidney disease (ESKD), the prevalence of LVH is particularly high, reaching 70%–80% (5, 6), and has been identified as an independent predictor of all-cause and cardiovascular mortality in those undergoing peritoneal dialysis (PD) (7–9). The pathogenesis of myocardial hypertrophy in dialysis patients is multifactorial, involving numerous pathophysiological processes and fundamentally reflecting an adaptive response to chronic pressure and volume overload (10). Hypertension plays a particularly pivotal role among these risk factors. It is not only highly prevalent in PD patients but also a core driver of increased cardiac pressure load and LVH development (11–13).

In recent years, fibroblast growth factor 23 (FGF23) and Klotho protein have emerged as novel biomarkers of significant interest. Klotho exists primarily in two forms: membrane-bound and soluble. The membrane-bound form, expressed in tissues such as the renal distal tubules, acts as an essential co-receptor for FGF23, enabling its regulation of phosphate and vitamin D metabolism—together constituting the classical FGF23–Klotho axis (14, 15). Soluble α-Klotho is generated mainly through proteolytic cleavage or alternative splicing of the membrane-bound form and is released into the circulation (16). Its functions extend beyond its role as an FGF23 co-receptor, encompassing broad anti-aging, antioxidant, anti-fibrotic, and endothelial protective effects, and it is recognized as an important circulating protective factor (17, 18).

As CKD progresses, renal expression and shedding of soluble α-Klotho decline markedly, resulting in reduced circulating levels (19). Concurrently, circulating FGF23 increases progressively due to phosphate retention and compensatory pathways. Consequently, CKD patients often exhibit a biochemical profile characterized by elevated FGF23 and reduced soluble Klotho (20). Evidence suggests that elevated circulating FGF23 may exert direct cardiotoxic effects, whereas insufficient soluble Klotho may impair intrinsic cardiovascular protective mechanisms (21, 22). However, systematic studies remain scarce on how serum soluble α-Klotho and FGF23 jointly influence left ventricular hypertrophy in the specific subpopulation of hypertensive PD patients, or whether an interaction exists between these two factors.

Therefore, this study focused on hypertensive PD patients. By measuring serum FGF23 and soluble α-Klotho levels concurrently, we aimed to (1) analyze their independent and combined associations with LVH, (2) evaluate their predictive value for LVH, and (3) explore potential non-linear dose–response relationships and subgroup differences with left ventricular mass index.

## 2 Methods

### 2.1 Study design and participants

This cross-sectional study enrolled patients from the Peritoneal Dialysis Center of Changshu Second People's Hospital between June 2022 and June 2023. Eligible participants were adults (≥18 years) who had undergone maintenance peritoneal dialysis for at least three months and had a documented diagnosis of hypertension (based on medical history, antihypertensive medication use, or measured blood pressure ≥140/90 mmHg). Additionally, patients were required to be clinically stable, with no hospitalizations, significant volume overload, or major infections within the four weeks prior to assessment.

Key exclusion criteria were: (1) conditions that could confound cardiac structural assessment (e.g., moderate-to-severe valvular heart disease or hypertrophic cardiomyopathy); (2) a recent (within 3 months) history of acute coronary syndrome, stroke, or heart failure requiring hospitalization; (3) comorbidities known to markedly affect bone metabolism or systemic inflammation, such as active malignancy, autoimmune diseases, surgical hyperparathyroidism, cirrhosis, or active infection; (4) prior kidney transplantation; (5) pregnancy or lactation; and (6) inability to complete the study procedures or provide informed consent.

Based on these criteria, 124 patients were included in the final analysis. The study protocol was approved by the Ethics Committee of Changshu Second People's Hospital (Approval No.: L2022047), and all participants provided written informed consent.

### 2.2 Demographic and clinical data collection

This study collected baseline demographic and clinical data through a combination of electronic medical record review and structured patient interviews. Information obtained included gender, age, duration of peritoneal dialysis, anthropometric measurements, history of hypertension and diabetes, and mean predialysis blood pressure recorded during hospitalization. Fasting blood samples were collected on the morning following admission, prior to any dialysis session. Routine biochemical parameters—including C-reactive protein, haemoglobin, renal function, electrolytes, albumin, lipid profile, blood glucose, glycated haemoglobin, parathyroid hormone, and thyroid-stimulating hormone—were analyzed using standardized assays in the hospital's accredited clinical laboratory. Dialysis adequacy was assessed by calculating weekly total Kt/V. Serum concentrations of soluble α-Klotho and FGF23 were measured using commercially available enzyme-linked immunosorbent assay (ELISA) kits (Suzhou Kelvin Biotech Co., Ltd., China). All assays were performed in strict accordance with the manufacturer's instructions, with duplicate wells and quality-control samples included to ensure reproducibility and reliability.

### 2.3 Echocardiographic examination

Comprehensive transthoracic echocardiography was conducted by a single senior cardiologist who was blinded to all patient data, utilizing a standardized ultrasound device (HY-M50, Wuxi Haiying Electronic Medical Co., Ltd.). Measurements were obtained following the contemporary recommendations of the American Society of Echocardiography. Key parameters included interventricular septal thickness, left ventricular internal diameter, and posterior wall thickness at end-diastole, as well as left ventricular ejection fraction. Each measurement was averaged over three cardiac cycles.

Left ventricular mass (LVM) was calculated using the Devereux formula: LVM(*g*) = 0.8 × { 1.04 × [(IVST + + LVPWT + LVDD)^3^ − LVD D^3^] } + 0.6. The LVM index (LVMI, g/m^2^) was obtained by indexing LVM to body surface area (BSA), which was computed via the Stevenson formula. Relative wall thickness (RWT) was calculated as (IVST + LVPWT)/LVDD. Left ventricular hypertrophy (LVH) was defined as an LVMI >115 g/m^2^ for men and >95 g/m^2^ for women. Participants were subsequently classified into four distinct left ventricular geometric patterns based on their LVMI and RWT, as detailed in Table 1.

**Table 1:** Left ventricular geometry classification.

LV geometry	LVMI(g/㎡)	RWT
NG(Normal geometry)	M ≤ 115; F ≤ 95	≤0.42
CR(Concentric remodeling)	M ≤ 115; F ≤ 95	>0.42
CH(Concentric hypertrophy)	M > 115; F > 95	>0.42
EH(Eccentric hypertrophy)	M > 115; F > 95	≤0.42

LVMI, left ventricular mass index; RWT, relative wall thickness; NG, normal geometry; CR, concentric remodeling; CH, concentric hypertrophy; EH, eccentric hypertrophy.

### 2.4 Statistical analysis

All statistical analyses were performed using R software (version 4.3.1). The normality of continuous variables was assessed with the Shapiro–Wilk test. Normally distributed data are presented as mean ± standard deviation and were compared using independent-samples *t*-tests or one-way ANOVA. Non-normally distributed data are expressed as median (interquartile range) and were compared using the Mann–Whitney *U* test or Kruskal–Wallis test. Categorical variables are reported as frequency (percentage) and were compared by chi-square or Fisher's exact test, as appropriate. A two-tailed *P* value <0.05 was considered statistically significant. For variables with missing values (<5% of all data), multiple imputation was performed via chained equations using the mice package, generating five imputed datasets; results were pooled according to Rubin's rules.

Given the limited sample size in the original four-category left ventricular geometry classification (NG, CR, CH, EH), which could lead to unstable parameter estimates, the primary outcome was simplified to a binary classification: left ventricular hypertrophy (LVH, comprising CH and EH) vs. non-LVH (nLVH, comprising NG and CR). To identify independent factors associated with LVH, multivariable logistic regression models were constructed. Covariates were selected based on univariate results (*P* < 0.05, Table 2) and clinical relevance. To separately evaluate the independent contributions of serum FGF23 and soluble α-Klotho, four nested models were built: Model 1 adjusted for baseline confounders (sex, systolic blood pressure, C–reactive protein, hemoglobin, serum calcium, and ejection fraction); Model 2 added FGF23 to Model 1; Model 3 added soluble α-Klotho to Model 1; and Model 4 (the fully adjusted model) included all variables from Model 1 plus both FGF23 and soluble α-Klotho. Improvements in model fit across nested models were assessed using likelihood-ratio tests. Variance inflation factors (VIF) were calculated for all continuous predictors, with VIF <5 indicating no severe multicollinearity. Model performance was evaluated using receiver operating characteristic (ROC) curves, decision curve analysis (DCA), and calibration plots to assess discrimination, clinical utility, and calibration, respectively.

**Table 2:** Baseline characteristics of PD patients with or without LVH.

Variables	All (*n* = 124)	LVH (*n* = 78)	nLVH (*n* = 46)	Statistics	*P* value
Gender				16.199	<0.001
Female	56 (45.16%)	46 (59.0%)	10 (21.7%)		
Male	68 (54.84%)	32 (41.0%)	36 (78.3%)		
Age (year)	60.18 ± 13.09	59.85 ± 13.85	60.74 ± 11.80	−0.366	0.715
PD_duration (months)	39.00 (23.00, 56.50)	39.00 (22.00, 51.25)	39.00 (29.50, 66.50)	−0.362	0.717
BMI (kg/m^2^)	22.79 ± 3.49	23.14 ± 3.83	22.18 ± 2.75	1.491	0.139
SBP (mmHg)	144.00 (127.25, 166.00)	149.50 (137.00, 169.00)	129.50 (116.50, 154.50)	−3.322	<0.001
DBP (mmHg)	82.85 ± 12.43	83.33 ± 11.70	82.04 ± 13.67	−0.557	0.579
Diabetes (%)				0.027	0.869
No	42 (33.9%)	26 (33.3%)	16 (34.8%)		
Yes	82 (66.1%)	52 (66.7%)	30 (65.2%)		
Kt/V	1.61 ± 0.38	1.65 ± 0.04	1.53 ± 0.05	−1.794	0.075
CRP	3.55 (1.13, 6.75)	4.50 (1.48, 7.45)	2.65 (0.78, 4.95)	−2.139	0.032
Hgb (g/L)	90.44 ± 21.33	87.18 ± 2.65	95.96 ± 2.35	2.250	0.026
BUN (mmol/L)	21.70 ± 7.67	22.04 ± 8.34	21.13 ± 6.40	0.640	0.523
CREA (*μ*mol/L)	1,035.45 ± 304.47	1,024.74 ± 312.93	1,053.61 ± 292.05	−0.508	0.612
UA (μmol/L)	398.44 ± 111.14	406.22 ± 117.23	385.26 ± 99.81	1.014	0.312
ALB (g/L)	29.00 ± 4.08	28.41 ± 4.41	30.01 ± 3.25	−2.151	0.330
K (mmol/L)	3.69 ± 0.62	3.66 ± 0.59	3.74 ± 0.68	−0.710	0.479
NA (mmol/L)	140.76 ± 3.18	141.05 ± 3.21	140.28 ± 3.08	1.309	0.193
CL (mmol/L)	100.40 ± 4.04	100.28 ± 3.94	100.62 ± 4.25	−0.451	0.652
*P* (mmol/L)	2.11 ± 0.21	2.10 ± 0.22	2.13 ± 0.21	−0.748	0.456
Ca (mmol/L)	1.68 ± 0.59	1.77 ± 0.63	1.52 ± 0.50	2.348	0.021
TG (mmol/L)	1.24 (0.96,1.81)	1.27 (0.92,1.80)	1.23 (0.97,2.06)	−0.070	0.944
LDL (mmol/L)	2.14 ± 0.67	2.10 ± 0.67	2.22 ± 0.68	−0.995	0.322
GLU (mmol/L)	4.61 (4.05, 5.43)	4.67 (4.03, 5.36)	4.55 (4.14, 6.26)	−0.455	0.649
PTH (pg/mL)	245.65 (164.25, 447.75)	233.20 (142.75, 447.65)	283.10 (191.35, 467.85)	−1.022	0.307
TSH (uIU/mL)	3.04 (1.99, 6.53)	2.93 (1.82, 4.73)	3.16 (2.32, 7.66)	−1.697	0.090
HbA1c (%)	5.60 (4.90, 6.10)	5.50 (4.70, 6.10)	5.70 (5.10, 6.23)	−1.644	0.100
FGF23 (pg/mL)	40.79 ± 3.95	41.27 ± 4.35	39.98 ± 3.04	1.772	0.079
α-Klotho (ng/mL)	14.93 ± 1.77	14.56 ± 1.91	15.57 ± 1.29	−3.178	0.002
LVMI (g/m^2^)	111.57 (99.92, 140.28)	132.97 (111.95, 162.74)	91.70 (81.14, 103.25)	−8.064	<0.001
EF (%)	62.60 ± 7.97	61.39 ± 9.34	64.66 ± 4.20	−2.240	0.027

LVH, left ventricular hypertrophy; nLVH, non-left ventricular hypertrophy; SBP, systolic blood pressure; DBP, diastolic blood pressure; CRP, C-reactive protein; Hgb, hemoglobin; BUN, blood urea nitrogen; CREA, creatinine; UA, uric acid; ALB, albumin; Ca, calcium; P, phosphorus; TG, triglycerides; LDL, low-density lipoprotein; GLU, glucose; PTH, parathyroid hormone; TSH, thyroid-stimulating hormone; HbA1c, glycated hemoglobin; FGF23, fibroblast growth factor 23; LVMI, left ventricular mass index; EF, ejection fraction.

Finally, restricted cubic splines (RCS) with four knots placed at default percentiles were applied to flexibly model the dose-response relationships of FGF23 and soluble α-Klotho (as continuous variables) with LVMI, while testing for nonlinearity. These analyses were further stratified by median age and sex to explore potential effect modification.

## 3 Results

### 3.1 Baseline characteristics of PD patients with or without LVH

During the study period, 124 peritoneal dialysis patients were enrolled and stratified by the presence of left ventricular hypertrophy into two groups: an LVH group (*n* = 78) and a non-LVH (nLVH) group (*n* = 46). The baseline characteristics of the cohorts are summarized in Table 2. Compared with the nLVH group, patients in the LVH group had a significantly higher proportion of females (59.0% vs. 21.7%, *P* < 0.001), along with elevated systolic blood pressure, C-reactive protein, and serum calcium levels (all *P* < 0.05). Conversely, hemoglobin and serum soluble α-Klotho levels were significantly lower in the LVH group (both *P* < 0.05). As expected, the median left ventricular mass index was higher in the LVH group than in the nLVH group [132.97 (111.95–162.74) g/m^2^ vs. 91.70 (81.14–103.25) g/m^2^, *P* < 0.001], whereas the mean left ventricular ejection fraction was lower (61.39% ± 9.34% vs. 64.66% ± 4.20%, *P* = 0.027). No significant intergroup differences were observed in age, dialysis vintage, body mass index, diabetes prevalence, weekly total Kt/V, or other routine biochemical parameters (all *P* > 0.05).

This study stratified left ventricular geometry into four groups according to echocardiographic criteria: normal geometry (NG, *n* = 17), concentric remodeling (CR, *n* = 29), concentric hypertrophy (CH, *n* = 48), and eccentric hypertrophy (EH, *n* = 30). Univariate analysis indicated statistically significant differences among these groups across several clinical and biochemical parameters (Table 3).

**Table 3:** Baseline characteristics by left ventricular geometric patterns.

Variables	NG (*n* = 17)	CR (*n* = 29)	CH (*n* = 48)	EH (*n* = 30)	Statistics	*P* value
Gender					22.995	<0.001
Female	1 (5.9%)	9 (31.0%)	24 (50.0%)	22 (73.3%)		
Male	16 (94.1%)	20 (69.0%)	24 (50.0%)	8 (26.7%)		
Age (year)	62.82 ± 7.06	59.52 ± 13.82	62.21 ± 14.55	56.07 ± 11.93	1.655	0.181
PD_duration (months)	34.88 ± 18.13	52.59 ± 31.43	41.71 ± 24.74	57.87 ± 9.86	2.265	0.084
BMI (kg/m^2^)	21.64 ± 2.94	22.50 ± 2.63	23.13 ± 3.68	23.16 ± 4.12	0.947	0.420
SBP (mmHg)	115.00 (110.00, 141.00)	143.00 (124.00, 154.00)	151.50 (144.00, 169.00)	139.50 (129.00, 170.00)	18.659	<0.001
DBP (mmHg)	77.88 ± 12.12	84.48 ± 14.13	81.75 ± 10.30	85.87 ± 13.43	1.824	0.146
Diabetes (%)					9.080	0.028
No	5 (29.4%)	11 (37.9%)	22 (45.8%)	4 (13.3%)		
Yes	12 (70.6%)	18 (62.1%)	26 (54.2%)	26 (86.7%)		
Kt/V	1.58 (1.36, 1.66)	1.56 (1.32, 1.84)	1.67 (1.36, 1.91)	1.49 (1.31, 1.82)	3.741	0.291
CRP	5.97 ± 8.16	3.94 ± 4.71	6.59 ± 8.48	6.55 ± 6.85	4.930	0.177
Hgb (g/L)	92.76 ± 17.04	97.83 ± 15.27	88.83 ± 20.28	84.53 ± 27.93	2.143	0.098
BUN (mmol/L)	23.34 ± 5.39	19.83 ± 6.67	20.90 ± 6.75	23.87 ± 10.27	1.847	0.142
CREA (μmol/L)	1,158.76 ± 271.07	991.97 ± 290.58	1,010.88 ± 288.43	1,046.93 ± 352.63	1.253	0.294
UA (μmol/L)	401.76 ± 74.15	375.59 ± 112.28	390.98 ± 108.48	430.60 ± 128.15	1.334	0.267
ALB (g/L)	29.08 ± 3.24	30.56 ± 3.19	27.86 ± 4.02	29.27 ± 4.92	2.823	0.042
K (mmol/L)	3.70 ± 0.55	3.77 ± 0.75	3.65 ± 0.60	3.68 ± 0.58	0.242	0.867
NA (mmol/L)	139.96 ± 3.86	140.47 ± 2.58	141.08 ± 3.05	141.01 ± 3.51	0.656	0.581
CL (mmol/L)	99.83 ± 2.82	101.08 ± 4.89	99.89 ± 3.56	100.89 ± 4.48	0.783	0.506
*P* (mmol/L)	2.09 ± 0.20	2.15 ± 0.22	2.08 ± 0.23	2.12 ± 0.20	0.591	0.622
Ca (mmol/L)	1.42 ± 0.63	1.57 ± 0.41	1.67 ± 0.44	1.94 ± 0.82	3.518	0.017
TG (mmol/L)	1.68 ± 1.57	1.66 ± 1.15	1.71 ± 1.43	1.38 ± 0.63	1.273	0.736
LDL (mmol/L)	2.25 ± 0.79	2.20 ± 0.61	2.23 ± 0.65	1.88 ± 0.67	2.096	0.104
GLU (mmol/L)	4.66 (4.12, 6.25)	4.54 (4.32, 5.45)	4.63 (4.04, 5.40)	4.67 (3.98, 5.29)	1.370	0.713
PTH (pg/mL)	258.10 (111.10, 326.70)	323.90 (207.60, 559.30)	230.85 (153.50, 398.40)	241.30 (101.80, 690.60)	3.518	0.318
TSH (uIU/mL)	3.15 (2.41, 7.65)	3.72 (1.45, 7.35)	2.21 (1.82, 5.50)	3.33 (1.93, 4.11)	3.328	0.344
HbA1c (%)	5.80 (5.10, 6.00)	5.60 (5.10, 6.60)	5.80 (4.95, 6.20)	4.90 (4.60, 5.90)	9.730	0.021
FGF23 (pg/mL)	39.14 ± 2.13	40.47 ± 3.41	42.24 ± 4.12	39.73 ± 4.32	4.212	0.007
α-Klotho (ng/mL)	15.29 ± 1.72	15.73 ± 0.95	14.98 ± 1.61	13.88 ± 2.18	6.517	<0.001
LVMI (g/m2)	89.85 (84.76, 92.31)	97.22 (75.55, 105.96)	140.24 (123.18, 165.32)	114.32 (101.68, 139.30)	72.959	<0.001
EF (%)	64.90 (59.40, 69.10)	64.90 (63.10, 66.10)	65.10 (60.00, 67.90)	58.30 (45.10, 67.90)	7.313	0.063

NG, normal geometry; CR, concentric remodeling; CH, concentric hypertrophy; EH, eccentric hypertrophy; LVH, left ventricular hypertrophy; nLVH, non-left ventricular hypertrophy; SBP, systolic blood pressure; DBP, diastolic blood pressure; CRP, C-reactive protein; Hgb, hemoglobin; BUN, blood urea nitrogen; CREA, creatinine; UA, uric acid; ALB, albumin; Ca, calcium; P, phosphorus; TG, triglycerides; LDL, low-density lipoprotein; GLU, glucose; PTH, parathyroid hormone; TSH, thyroid-stimulating hormone; HbA1c, glycated hemoglobin; FGF23, fibroblast growth factor 23; LVMI, left ventricular mass index; EF, ejection fraction.

**Figure 1:**
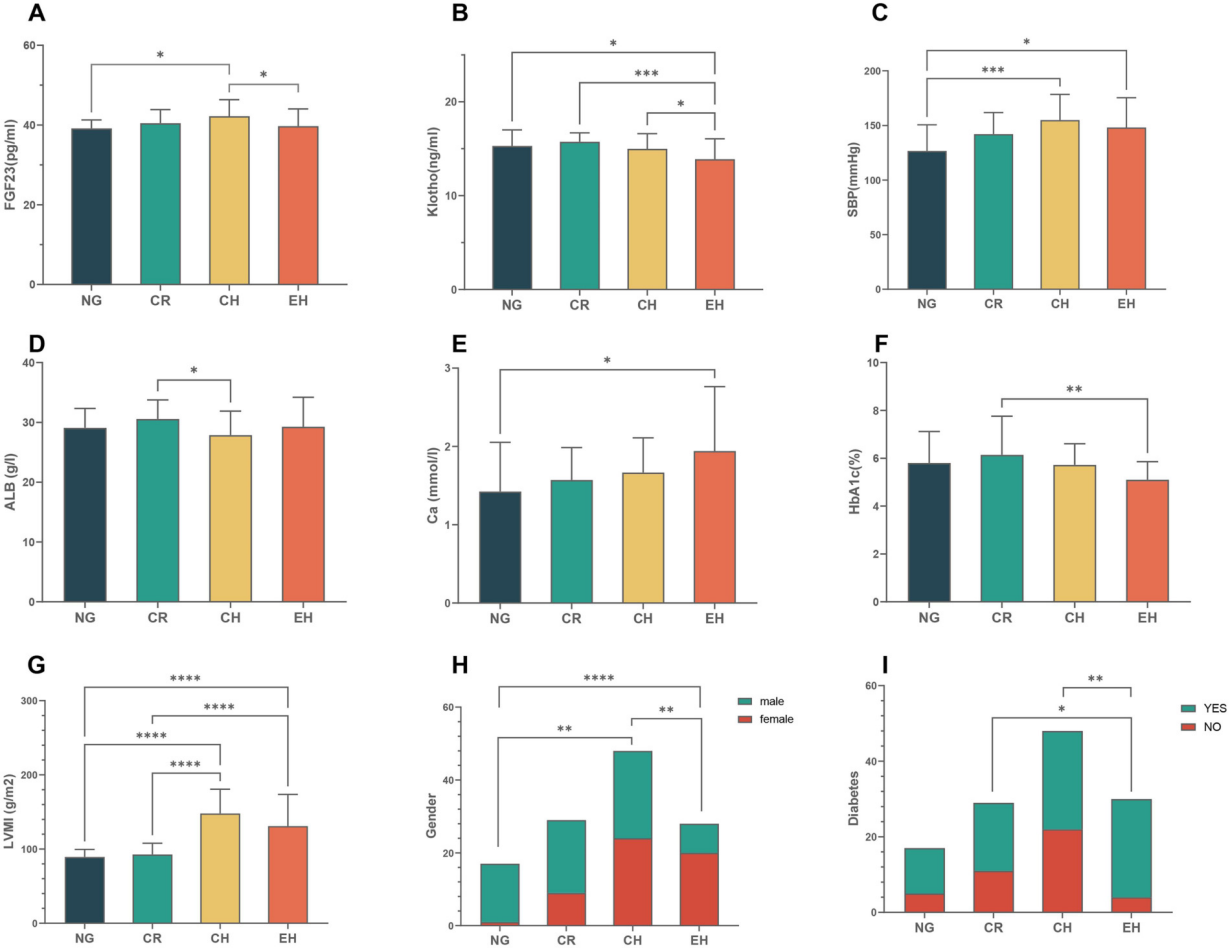
Intergroup differences of relevant indicators among different left ventricular structures. **(A)** FGF23, **(B)** α-Klotho, **(C)** SBP, **(D)** ALB, **(E)** Ca, **(F)** HbA1c, **(G)** LVMI, **(H)** Gender, **(I)** Diabetes.* *P* ≤ 0.05;** *P* ≤ 0.01;*** *P* ≤ 0.001;**** *P* ≤ 0.0001.

Specifically, intergroup differences were significant for sex, systolic blood pressure (SBP), prevalence of diabetes, and levels of albumin (ALB), serum calcium (Ca), glycated hemoglobin (HbA1c), FGF23, and soluble α-Klotho (all *P* < 0.05). *Post-hoc* comparisons showed that serum FGF23 was significantly higher in the CH group than in the NG and EH groups, while soluble α-Klotho was significantly lower in the EH group compared with the other three groups (Figures 1A,B). Systolic blood pressure was lower in the NG group than in the CH and EH groups. A significant difference in ALB was observed between the CR and CH groups (*P* < 0.05). Notably, despite having the highest proportion of females and the highest prevalence of diabetes, the EH group exhibited the lowest HbA1c levels among all groups, along with the highest circulating levels of Ca and FGF23 (Figures 1C–I).

Left ventricular mass index (LVMI) differed markedly across groups (*P* < 0.001). Pairwise comparisons revealed no significant difference in LVMI between the CH and EH groups; however, both groups had significantly higher LVMI than the NG and CR groups, with the CH group showing the highest median value. No significant intergroup differences were detected in age, dialysis vintage, weekly total Kt/V, or other routine biochemical measures (all *P* > 0.05).

### 3.3 Independent factors associated with left ventricular hypertrophy and model performance

Using non-left ventricular hypertrophy (nLVH) as the reference, multivariable logistic regression was performed to identify factors independently associated with LVH. As shown in Table 4, after adjustment for sex, systolic blood pressure (SBP), C–reactive protein (CRP), hemoglobin (Hgb), serum calcium (Ca), and ejection fraction (EF) in Model 1, male sex was a strong protective factor (OR = 0.116, 95% CI: 0.037–0.314, *P* < 0.001), whereas higher SBP (OR = 1.041, 95% CI: 1.017–1.069, *P* = 0.001), lower Hgb (OR = 0.974, 95% CI: 0.951–0.996, *P* = 0.022), and lower EF (OR = 0.906, 95% CI: 0.818–0.983, *P* = 0.035) were independently associated with increased risk of LVH.

**Table 4:** Logistic regression model (reference: nLVH).

Characteristics	Model 1		Model 2		Model 3		Model 4	
	OR(95%CI)	*P* value	OR(95%CI)	*P* value	OR(95%CI)	*P* value	OR(95%CI)	*P* value
Gender (reference = Female)	0.116 (0.037–0.314)	<0.001	0.116 (0.037–0.315)	<0.001	0.071 (0.018–0.222)	<0.001	0.056 (0.013–0.188)	<0.001
SBP (Continuous)	1.041 (1.017–1.069)	0.001	1.041 (1.017–1.071)	0.002	1.038 (1.014–1.067)	0.004	1.04 (1.011–1.074)	0.010
CRP (Continuous)	1.047 (0.982–1.128)	0.188	1.05 (0.983–1.134)	0.177	1.025 (0.961–1.103)	0.474	1.017 (0.947–1.103)	0.653
Hgb (Continuous)	0.974 (0.951–0.996)	0.022	0.977 (0.954–1)	0.053	0.958 (0.93–0.984)	0.003	0.964 (0.936–0.989)	0.008
Ca (Continuous)	1.779 (0.741–4.685)	0.215	1.806 (0.743–4.849)	0.211	1.068 (0.42–2.893)	0.892	0.909 (0.336–2.601)	0.853
EF (Continuous)	0.906 (0.818–0.983)	0.035	0.901 (0.811–0.981)	0.032	0.898 (0.806–0.975)	0.025	0.87 (0.763–0.959)	0.016
FGF23 (Continuous)	–	–	1.063 (0.945–1.202)	0.312	–	–	1.26 (1.079–1.501)	0.005
α-Klotho (Continuous)	–	–	–	–	0.585 (0.398–0.822)	0.003	0.415 (0.247–0.643)	<0.001
AIC	126.98		127.94		119.01		112.17	
BIC	146.72		150.50		141.57		137.55	

Model 1: Adjusted for Gender, SBP, CRP, Hgb, Ca, EF. Model 2: Model 1 + FGF23. Model 3: Model 1 +α-Klotho. Model 4: Model 1 + FGF23 +α-Klotho (Fully adjusted). OR, odds ratio; CI, confidence interval.

When FGF23 was added to Model 1 (Model 2), FGF23 itself was not significantly associated with LVH (OR = 1.063, *P* = 0.312). In contrast, soluble α-Klotho remained an independent protective factor in Model 3 (OR = 0.585, 95% CI: 0.398–0.822, *P* = 0.003). The fully adjusted model (Model 4), which included both FGF23 and soluble α-Klotho, showed improved AIC and BIC values compared with the other models. In this final model, soluble α-Klotho retained a strong protective effect (OR = 0.415, 95% CI: 0.247–0.643, *P* < 0.001), while FGF23 emerged as an independent risk factor (OR = 1.260, 95% CI: 1.079–1.501, *P* = 0.005).

**Figure 2:**
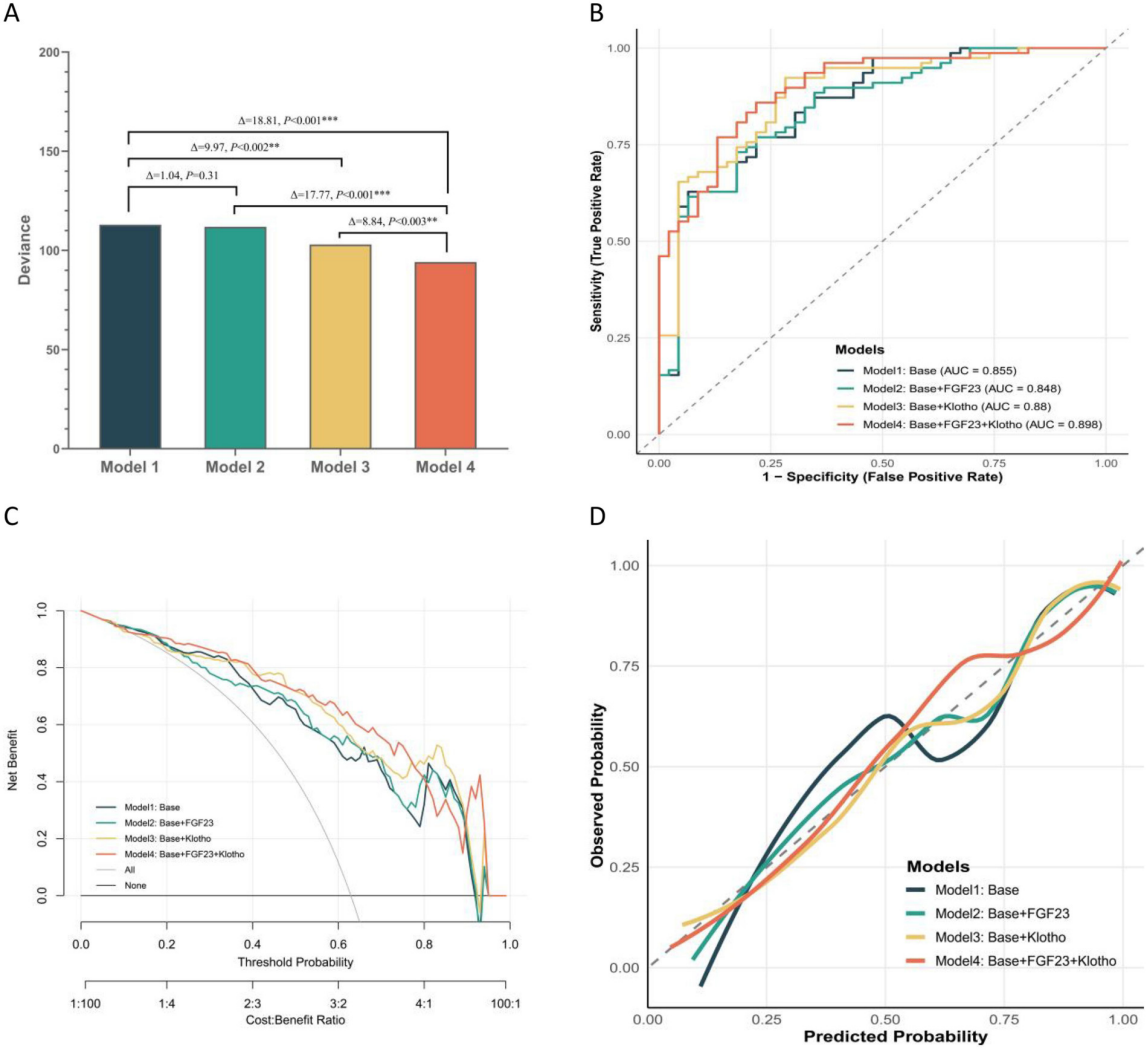
Model performance analysis. **(A)** Nested model likelihood ratio test results (* *P* < 0.05, ** *P* < 0.01, *** *P* < 0.001); **(B)** receiver operating characteristic (ROC) curves for the four models; **(C)** decision curve analysis (DCA) for the four models; **(D)** calibration curves for the four models.

Likelihood-ratio tests (Figure 2A) confirmed that adding soluble α-Klotho significantly improved model fit (ΔDeviance = 9.97, *P* = 0.002), and the inclusion of both biomarkers provided the greatest improvement (ΔDeviance = 18.81, *P* < 0.001). Moreover, adding soluble α-Klotho to a model already containing FGF23 (Model 2 vs. Model 4), or adding FGF23 to a model already containing α-Klotho (Model 3 vs. Model 4), each contributed significant additional predictive information. ROC analysis (Figure 2B) indicated that Model 4 achieved the largest area under the curve (AUC = 0.898). Decision-curve analysis (Figure 2C) showed that Model 4 provided greater clinical net benefit across a wide range of threshold probabilities. Calibration curves (Figure 2D) demonstrated good agreement between predicted and observed probabilities for Model 4.

### 3.4 Nonlinear associations of FGF23 and α-Klotho with LVMI and stratified analyses

**Figure 3:**
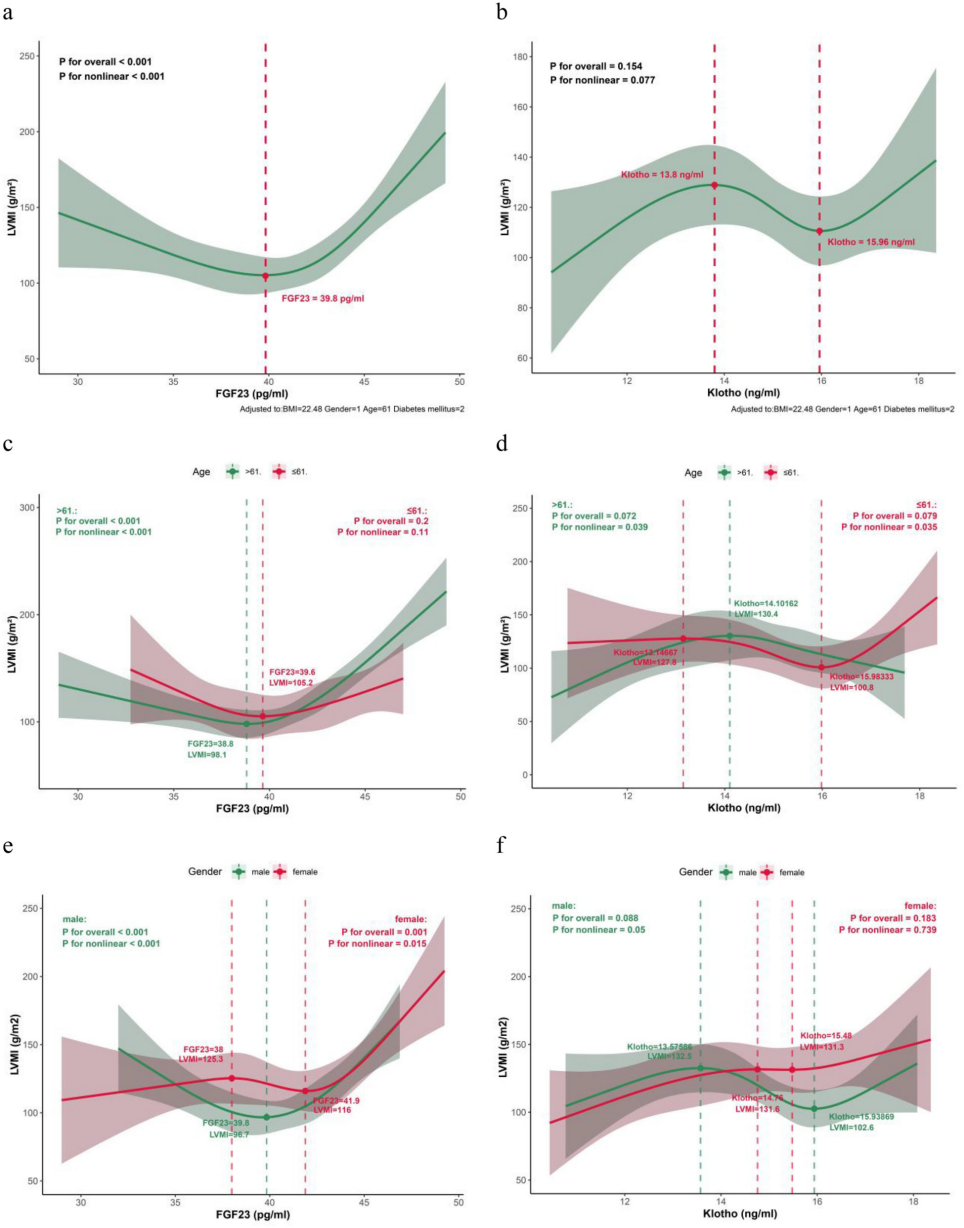
Restricted cubic spline (RCS) plot of FGF23, α-Klotho, and LVMI. **(a)** FGF23 vs. LVMI (overall). **(b)** α-Klotho vs. LVMI (overall). **(c)** FGF23 vs. LVMI stratified by age. **(d)** α-Klotho vs. LVMI stratified by age. **(e)** FGF23 vs. LVMI stratified by sex. **(f)** α-Klotho vs. LVMI stratified by sex.

After adjustment for body mass index, sex, age, and diabetes status, restricted cubic spline analysis demonstrated a significant nonlinear relationship between serum FGF23 and left ventricular mass index (overall *P* < 0.001, nonlinear *P* < 0.001). An inflection point was observed at approximately 39.8 pg/mL; beyond this threshold, LVMI rose steeply with increasing FGF23 (Figure 3a). In contrast, the overall association between soluble α-Klotho and LVMI was not statistically significant (overall *P* = 0.154), although a nonlinear trend was suggested (nonlinear *P* = 0.077) (Figure 3b). Because tests for nonlinearity indicated age as a significant effect modifier, further analyses were stratified by age and sex.

Age stratification showed that the strong nonlinear association between FGF23 and LVMI was confined to the older subgroup (>61 years; overall and nonlinear *P* < 0.001), with a threshold around 39 pg/mL (Figure 3c). No such association was evident in younger patients (≤61 years). For α-Klotho, statistically significant nonlinear relationships with LVMI were detected in both age groups (nonlinear *P* < 0.05 in each). Notably, in older patients, LVMI declined markedly once α-Klotho levels exceeded approximately 14.1 ng/mL (Figure 3d).

Sex-stratified analyses (Figures 3e,f) revealed that the positive correlation between FGF23 and LVMI remained significant in both men and women (overall *P* < 0.001 and *P* = 0.001, respectively), although the slope of increase was steeper in men. The relationship between α-Klotho and LVMI, however, differed by sex. A significant nonlinear pattern was observed in men (nonlinearity *P* = 0.05), characterized by an initial decline in LVMI as α-Klotho reached about 13.6 ng/mL, followed by a rebound above 15.5 ng/mL. This complex, non-monotonic association was not present in women.

## 4 Discussion

In this cross-sectional study, we systematically evaluated the association of serum FGF23 and soluble α-Klotho with left ventricular hypertrophy (LVH) in hypertensive peritoneal dialysis patients. The results indicate that soluble α-Klotho serves as an independent protective factor against LVH, whereas elevated FGF23 levels are independently associated with increased LVH risk after adjustment for α-Klotho. Furthermore, both biomarkers showed complex non-linear relationships with left ventricular mass index, with variations across different demographic subgroups.

### 4.1 Factors associated with left ventricular hypertrophy

This study identified female sex, elevated systolic blood pressure, increased C-reactive protein and serum calcium levels, as well as reduced hemoglobin, ejection fraction, and serum soluble α-Klotho levels as significant factors associated with LVH in hypertensive peritoneal dialysis patients. These findings align partially with both traditional and emerging concepts of cardiovascular complications in CKD.

Beyond conventional risk factors, the unique pathophysiological milieu of dialysis patients represents a critical driver of left ventricular remodeling. Intergroup comparisons in this study offer insights into the features of distinct remodeling patterns. Volume overload and inadequate blood pressure control are established contributors to eccentric hypertrophy (EH). In our cohort, both the EH and concentric hypertrophy (CH) groups had significantly higher systolic pressure than the normal geometry (NG) group, with no statistically significant difference in LVMI between EH and CH, underscoring the central role of pressure overload in both phenotypes (23). The EH group also exhibited the lowest soluble α-Klotho levels alongside the highest serum calcium and FGF23 concentrations, suggesting more pronounced α-Klotho deficiency, mineral metabolism disturbance, and possibly a greater burden of underlying vascular calcification. These factors may promote left ventricular chamber dilation and eccentric remodeling independently of pressure overload.

Concentric hypertrophy (CH) may reflect more marked metabolic and hormonal dysregulation. The CH group showed the highest FGF23 levels, significantly exceeding those in the NG and EH groups. This implies a potential distinct role for FGF23 in promoting cardiomyocyte hypertrophy and interstitial fibrosis, possibly via pathways modulated when α-Klotho is relatively preserved (compared to EH). Additionally, the difference in albumin between the CH and concentric remodeling (CR) groups suggests that the nutrition–inflammation axis may differentially influence various stages of concentric remodeling (24).

Our analysis also revealed metabolic dissociation across geometric subgroups. Notably, despite having the highest prevalence of diabetes, the EH group displayed the lowest HbA1c levels. This may reflect altered glucose metabolism following progression from diabetic nephropathy to end-stage renal disease, highlighting the complex metabolic adaptations in this population (25, 26). Furthermore, neither dialysis vintage nor Kt/V differed significantly across groups, indicating that these parameters may not be primary determinants of left ventricular geometry in this cohort, although their pervasive influence as background pathological factors remains.

Sex differences significantly influenced LVH development. Among hypertensive patients, females demonstrate greater susceptibility to LVH and left atrial enlargement than males, though underlying mechanisms are not fully understood. One report noted a higher prevalence of overall LVH and concentric hypertrophy in females compared with males, with no significant sex difference for eccentric hypertrophy (27). In our study, not only was the proportion of females higher in the overall LVH group, but both the CH and EH subgroups contained a greater proportion of females than the NG subgroup. Moreover, the sex distribution differed significantly between the CH and EH subgroups (*P* < 0.01).

### 4.2 Correlation between serum FGF23 and soluble α-Klotho levels and left ventricular hypertrophy

A key finding of this study is that, after adjustment for multiple confounders including inflammation, anemia, and calcium-phosphorus metabolism, serum soluble α-Klotho remained independently and inversely associated with LVH risk (Model 4: OR = 0.415, *P* < 0.001). This suggests that soluble α-Klotho may exert a cardioprotective effect in dialysis patients that extends beyond conventional risk factors, potentially through mechanisms involving attenuation of myocardial oxidative stress and apoptosis, suppression of fibrosis, and improvement of endothelial function.

Although FGF23 did not show significant intergroup differences in univariate analysis, it emerged as an independent risk factor for LVH after adjusting for α-Klotho in the multivariable model (Model 4: OR = 1.260, *P* = 0.005). Of note, the association of FGF23 with LVH was weaker and non-significant in models that did not include α-Klotho (Model 2), but became stronger and statistically significant in the fully adjusted model. This pattern suggests that α-Klotho may act as an important confounder or effect modifier: higher α-Klotho levels likely coincide with lower FGF23 and reduced LVH risk, thereby partially masking the independent contribution of FGF23 when α-Klotho is not accounted for. These observations imply that the adverse cardiac effects of FGF23 may be unmasked or amplified under conditions of relative α-Klotho deficiency. This supports the concept that “Klotho deficiency predisposes to off-target cardiovascular toxicity of FGF23” (28, 29), underscoring the necessity of considering endogenous α-Klotho levels when evaluating FGF23-associated cardiovascular risk in dialysis patients.

### 4.3 Serum FGF23 and soluble α-Klotho levels exhibit a non-linear dose-response relationship with LVMI

RCS analysis demonstrated a significant nonlinear relationship between FGF23 and left ventricular mass index (overall *P* < 0.001, nonlinear *P* < 0.001), with an apparent inflection point at approximately 39.8 pg/mL. Beyond this level, LVMI increased sharply, suggesting a potential “critical point” in FGF23-related cardiac effects. It should be noted, however, that this observation arises from modeling within a relatively small cross-sectional sample; the identified inflection point lies near the upper reference limit of intact FGF23 in healthy individuals and should not be regarded as a general biological threshold or clinical intervention cutoff.

Furthermore, subgroup analyses revealed the modifying effects of age and sex on the association between serum FGF23 and soluble α-Klotho levels. Age-stratified analysis revealed that the nonlinear association of FGF23 with LVMI was significant only in older patients (>61 years; overall and nonlinear *P* < 0.001). In contrast, α-Klotho showed a significant nonlinear association with LVMI in both younger (≤61 years) and older subgroups (nonlinear *P* < 0.05 in both), with a notable decline in LVMI when α-Klotho exceeded about 14.1 ng/mL in the older cohort. These findings suggest that elderly peritoneal dialysis patients may be particularly susceptible to elevated FGF23 levels.

Sex-stratified analysis indicated that the positive correlation between FGF23 and LVMI remained significant in both men and women (overall *P* < 0.001 and *P* = 0.001, respectively), although the slope of increase was steeper in men. The relationship between α-Klotho and LVMI, however, displayed a significant nonlinear pattern only in males (nonlinearity *P* = 0.05), characterized by an initial decrease in LVMI as α-Klotho rose to approximately 13.6 ng/mL, followed by a rebound above 15.5 ng/mL. This sex-specific pattern may be related to potential cardioprotective effects of estrogen, which could attenuate FGF23-mediated toxicity by downregulating myocardial receptor expression (27).

## 5 Limitations

This study has several limitations. Firstly, the sample size was relatively small (*n* = 124) and derived from a single-centre retrospective cohort, potentially introducing selection bias (e.g., exclusion of patients with malignancies or severe infections). Consequently, the generalisability of these findings requires validation through larger-scale, multicentre prospective studies. Secondly, although we observed associations between FGF23 and α-Klotho with left ventricular remodelling, the underlying molecular mechanisms remain unexplained and require further investigation through basic research. Thirdly, despite adjusting for several confounding factors, residual confounding (such as volume load, dialysate characteristics, or specific medication use) may still exist.

## 6 Conclusion

This cross-sectional study confirms that serum soluble α-Klotho serves as an independent protective factor against left ventricular hypertrophy (LVH) in hypertensive peritoneal dialysis patients, whilst FGF23 constitutes an independent risk factor. Together, they form a crucial biomarker combination for assessing cardiac remodelling risk. Further findings reveal distinct pathophysiological characteristics corresponding to different left ventricular geometries: eccentric hypertrophy correlates with more pronounced α-Klotho deficiency and calcium-phosphorus metabolism disorders, whereas concentric hypertrophy is more closely associated with elevated FGF23 levels. Moreover, a non-linear relationship exists between FGF23 and LV mass index, with its influence modified by age and sex. These findings suggest that concurrent monitoring of FGF23 and soluble α-Klotho in clinical management may aid early identification of high-risk cardiac remodelling phenotypes, providing a basis for future exploration of cardioprotective intervention strategies based on this combined biomarker.

## Data Availability

The raw data supporting the conclusions of this article will be made available by the authors, without undue reservation.
